# Beyond the shell: malacology in medical dermatology

**DOI:** 10.1007/s00403-024-03343-z

**Published:** 2024-08-24

**Authors:** Max Oscherwitz, Brandon M. Godinich, Nupur Singh, Bethany R. Rohr

**Affiliations:** 1 Center for Dermatology Research, Department of Dermatology, Wake Forest School of Medicine, Winston-Salem, NC 27157-1071 USA; 2 Texas Tech Health Science Center El Paso Paul L. Foster School of Medicine, El Paso, TX USA; 3 University of Tennessee Health Science Center, Memphis, TN USA; 4 Department of Dermatology, University Hospitals Cleveland Medical Center and Case Western Reserve University School of Medicine, Cleveland, OH USA

**Keywords:** Malacology, Dermato-malacology, Mollusks, Snail Mucin

## Classification of mollusks and introduction to malacology

Mollusks are soft-bodied organisms that comprise the second-largest phylum of invertebrate animals [1]. Examples of mollusks include snails and slugs (*gastropods*), octopi and squid (*cephalopods*), and shelled scallops, mussels, clams, and oysters (*bivalves*). While historically renowned for their ecological significance and culinary delights, mollusks have increasingly become subjects of interest in medical research.

Malacology, the scientific study of mollusks, has helped highlight the rich repository of bioactive compounds and therapeutic agents that hold promise for medical applications [2]. Since ancient times, mollusk extracts have been used for various therapeutic effects, providing an alternative to opioid-based pain treatments, treating fevers, and alleviating respiratory ailments, including tuberculosis, pneumonia, asthma, bronchitis, and COVID-19 [3, 4].

This paper aims to delve into the multifaceted world of dermato-malacology, exploring its potential applications in medical dermatology and elucidating its mechanisms of action in wound healing, cancer therapy, and beyond. Of note, the main goal of our paper is to educate clinicians, patients, and trainees about the potential of dermato-malacology. Many of the therapeutic options discussed are still in development, but to our knowledge, a detailed discussion of dermato-malacology has not previously been conducted.

To accomplish this, we conducted a literature search for articles. Inclusion criteria included articles that were published between 2005 and 2024, and that focused on medical uses for gastropods, cephalopods, and bivalves with relevance to medical dermatology. Exclusion criteria included applications not relevant to human applications of malacology (i.e. veterinary care, marine biology, etc…).

## Use of gastropods in Dermatology

**Fig. 1:**
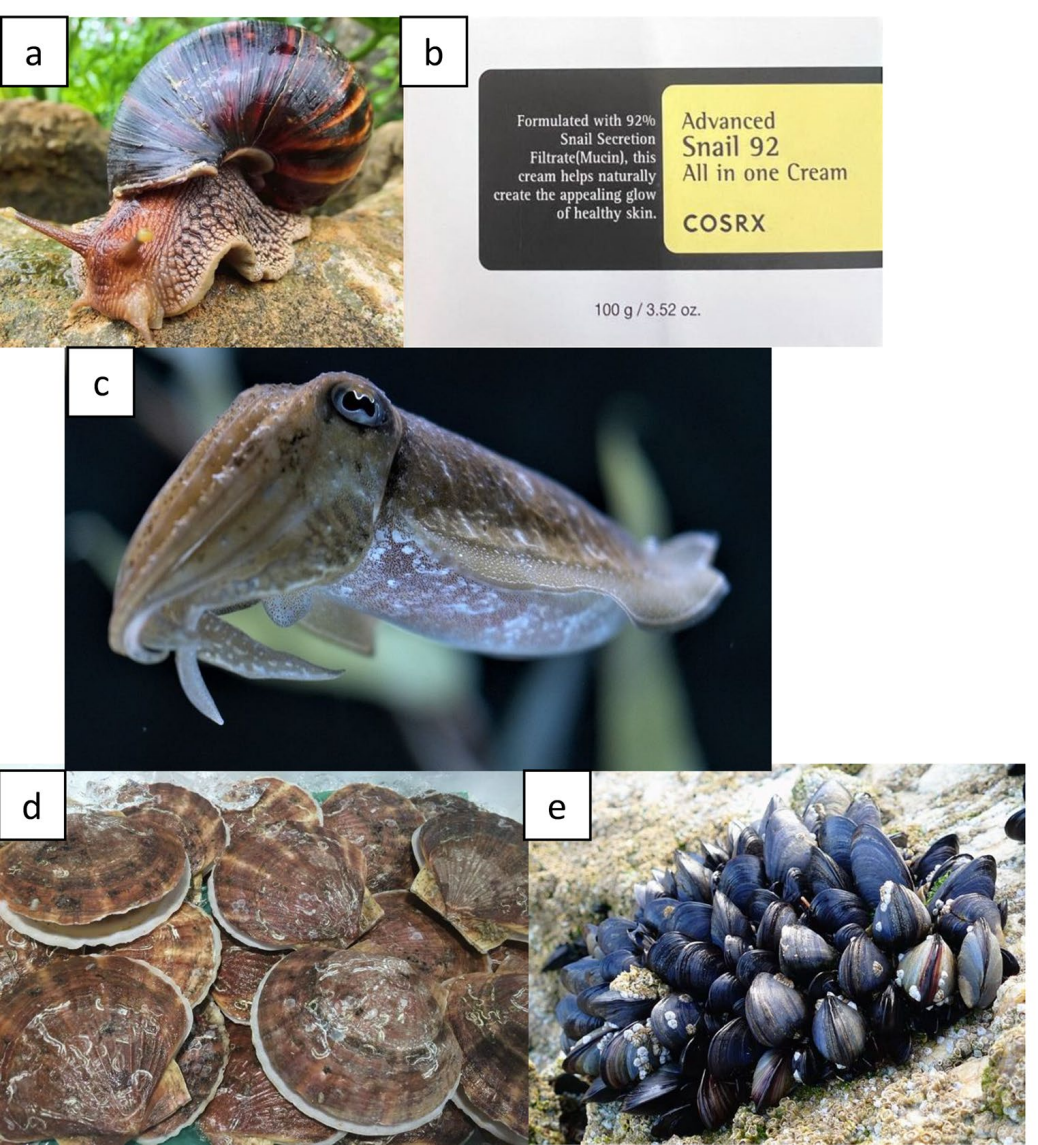
**(a-e): a**: A photo of a giant African snail (Achatina fulica), a species commonly used to produce snail mucin for commercial products. **b**: An example of snail-mucin products that are commercially available in retail stores. **c**: A photo of a cuttlefish (Sepia), which is a subtype of cephalopod whose ink can be used for artistic, culinary, and medicinal purposes. **d**: A photo of Yesso scallops (*Mizuhopecten yessoensis*). These commonly consumed mollusks may reduce photodamage.**e**: A photo demonstrating blue sea mussels (*Mytilus edulis*), which are commonly found in marine environments. These organisms are currently being investigated for their ability to mitigate scarring. *Figures 1a, 1c, 1d, and 1e used in accordance with the Content License Summary of Pixabay

Concentrated gastropod mucins are an emerging research topic in cosmetic and medical dermatology for several reasons. Over the past decade, social media endorsements have helped fuel the market for snail mucin to a global value of 1.56 billion USD, with an anticipated increase to 2.93 billion USD by 2034 [5]. Several extracts from the garden snail (*Helix aspersa*), burgundy snail (*Helix pomatia*), banana rasp snail (*Archachatina marginata*), kalutara snail (*Achatina fulica*), and giant African snail (*Achatina fulica*) are actively patented for their therapeutic effects, and their extracts are widely commercially available. (Figure 1a and b) [6]. A diverse array of bioactive molecules, including lactic, hyaluronic, and glycolic acid, and proteins, such as collagen and elastin, comprise snail mucin [7, 8]. These compounds are heralded for their cosmetic (i.e., anti-aging and hydrating) effects, but they also possess valuable properties for medical dermatologic conditions [7].

The wound-healing properties of snail mucin have long been recognized in ancient medicine, where snail secretions were used to promote the healing of cuts, burns, and abrasions [6]. Modern scientific research has provided insights into the mechanisms underlying the wound-healing activity of snail mucin, revealing its ability to accelerate tissue regeneration and enhance the repair process in an in vivo mice model [9]. The complex mixture of growth factors, cytokines, and extracellular matrix components found in snail mucin promotes cell proliferation, migration, and differentiation, facilitating the formation of new tissue and the closure of wounds [9]. Moreover, hyaluronic acid, a significant component of snail mucin, is crucial in maintaining tissue hydration and promoting a moist wound environment conducive to healing. Clinical studies of mice have demonstrated the efficacy of snail mucin-based formulations in promoting wound healing and reducing scar formation in various dermatological conditions, including burns, diabetic ulcers, and surgical wounds [10]. In modern times, snail mucin is heralded for its ability to provide hydration, combat hyperpigmentation, breakouts of acne, and potential scarring.

Snail mucin’s antimicrobial peptides and proteins provide broad-spectrum antimicrobial activity against a wide range of pathogens, including bacteria, viruses, protozoa, and fungi. The extract has been shown to possess antiviral properties, inhibiting the replication of viruses such as herpes simplex virus (HSV) and human papillomavirus (HPV). Its antiprotozoal activity has been investigated for the treatment of parasitic infections such as leishmaniasis and malaria. Additionally, snail mucin demonstrates antifungal activity against common dermatophytes and yeast species in vitro, making it a potential therapeutic agent for dermatophyte skin infections [6, 11].

Finally, from an oncologic perspective, snail mucin may target melanoma. In vitro studies in mice have demonstrated that the substance can decrease melanogenesis by inducing apoptosis and actively inhibiting the migration and invasion of melanoma cells by mediating metalloproteinase and integrin activity [7, 12, 13].

## Use of cephalopods in dermatology

Cephalopods are intelligent organisms often found in the dark depths of marine environments; however, they have potential to treat complex cutaneous ailments. Aside from their uses in culinary and artistic fields, medical products sourced from squid and cuttlefish inks have risen in popularity over the decades [14]. Unlike gastropods, there does not appear to be a common species routinely used to produce dermatologic products or whose products have been certified for widespread clinical use.

**Fig. 2:**
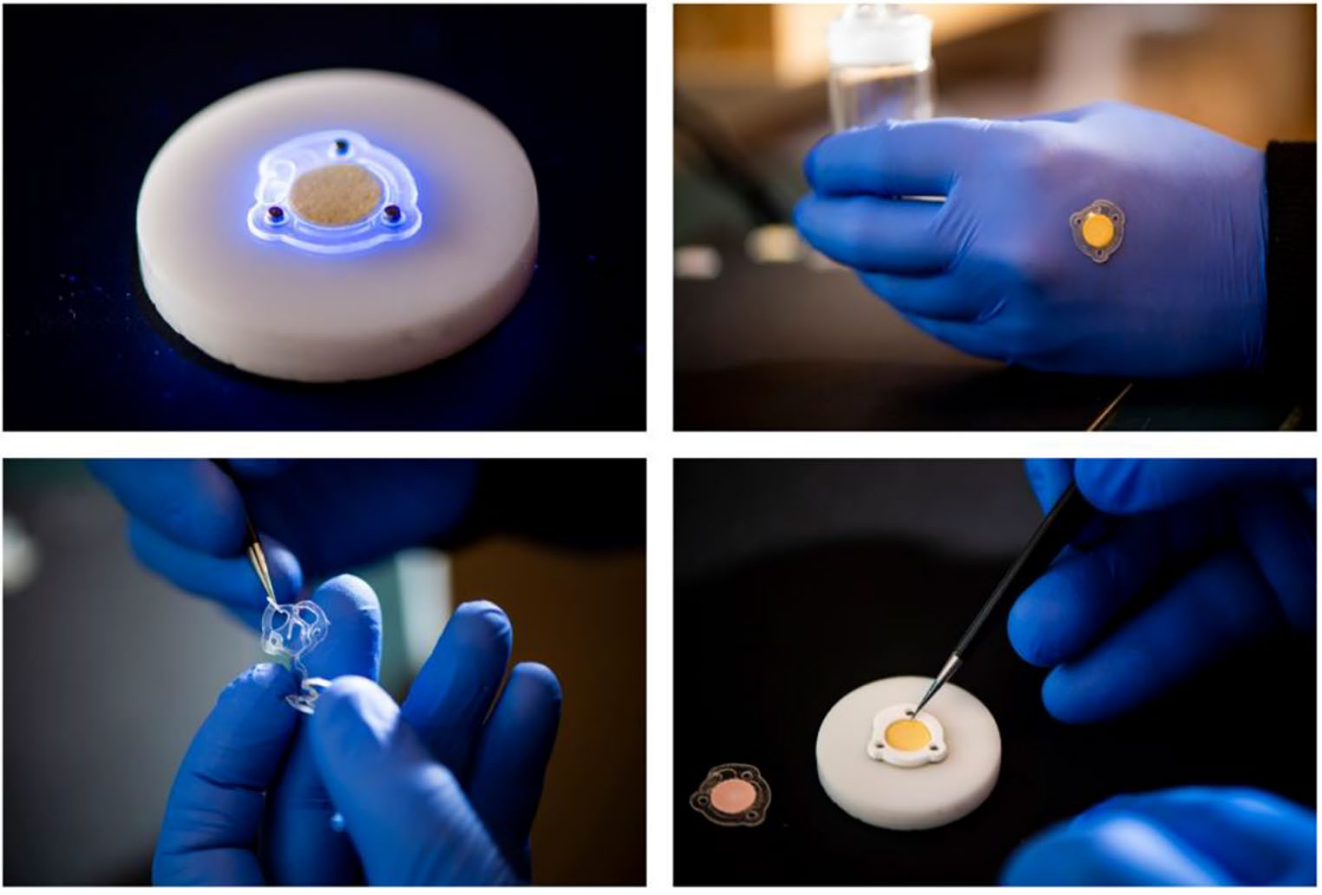
Photographs depicting how a sensor crafted based on the chemistry of squid ink functions by utilizing xanthommatin. When ultraviolet light reaches the sensor, it changes colors from yellow to red. This wearable device may warn users when they receive excess ultraviolet radiation after sun exposure. Photos credited to Alyssa Stone. Reproduced with permission from Dan Wilson

The chromatophores of cephalopods contain xanthommatin, a color-changing pigment that allows these organisms to camouflage within their surroundings. Xanthommatin is unique because it reacts to infrared and ultraviolet (UV) radiation. Scientists have explored xanthommatin’s potential for dermatologic application and have utilized the pigment to craft a pressure-activated microfluidic system that serves as a wearable light sensor (Fig. 2) [15]. Though not yet commercially available, this cephalopod-based invention may allow users to track and quantify their exposure to potentially harmful radiation.

Cephalopod derivatives may eventually be a therapeutic option for melanoma. In a pre-clinical trial, tachykinin-peptide (Octpep-1) from the southern sand octopus (*Octopus kaurna*) demonstrated the ability to reduce the progression of melanoma. The tachykinin-peptide induced cytotoxicity of malignant melanocytes in mice and zebrafish models [16]. Additionally, cuttlefish (*Sepiella maindroni* and *Sepia esculenta*) ink extracts can suppress melanoma and adenocarcinoma proliferation in vitro, although these observations have not yet been tested clinically. (Fig. 1c) [17].

Finally, squid-inspired products may provide novel clues into wound healing and skin regeneration. Isolated collagen grafts and their purified analogs from the Indian Ocean iris squid (*Sthenoteuthis oualaniensis*) have been tested in isolated and in combination peptides to determine their effect on the molecular pathogenesis of wound healing in a model built fram purified iris squid skin and synthesized collagen grafts [18]. Though purely experimental in vitro, via manipulation of the PI3 kinase/AKT and Ras/RAF/MAP kinase signaling pathways, these extracts enhanced the wound-healing activity of fibroblasts, theoretically making them a future avenue for treatment [19]. Furthermore, collagen scaffolds produced from processed Humboldt squid (*Dosidicus gigas*) have shown in vitro promise as a biodegradable and non-cytotoxic option to artificial collagen scaffolds for promoting tissue regeneration [18].

## Use of bivalves in dermatology

Bivalve mollusks are a diverse group of primitive yet efficient organisms characterized by an outer shell covering [20]. These organisms are vital for the health of ecosystems, as they filter sediments and serve as a food source for larger aquatic and terrestrial predators. Though not as widely studied as gastropods and cephalopods, the high prevalence of bioactive compounds in bivalves and their shells makes them attractive targets for dermatologic use.

Mussels are commonly consumed in Eastern Asian cuisine; however, their outer shells have few uses. Japanese scientists assessed the effect of shell extract obtained from the widely consumed Yesso scallop (*Mizuhopecten yessoensis*) on combatting cellular damage induced by UV-B light. (Fig. 1d) This in vivo experiment resulted in the proliferation and recapitulation of the epidermis, representing a potential agent to combat solar damage during in vivo rodent studies [21].

The prevalence of polysaccharides in the outer shells of bivalves makes their extracts an attractive target for wound healing. A Chinese research team emulated the amino acid-rich molecular secretions utilized by various mussel (Mytilidae) species to adhere to their environments to create a synthetic injectable hydrogel [22]. The hydrogel displayed superior tissue-anchoring properties compared to fibrin glue, especially under wet laboratory conditions [22]. The injectable substance demonstrated hemostatic abilities and exhibited antibacterial activity against *E. coli* and *S. aureus*, further highlighting its potential use in various aspects of wound healing (Fig. 1e) [22]. Because mussel-inspired bioadhesives are biodegradable and do not result in an inflammatory process as significantly as traditional sutures, they may provide an eco-friendly and efficacious sutureless wound closure technique [23].

**Fig. 3:**
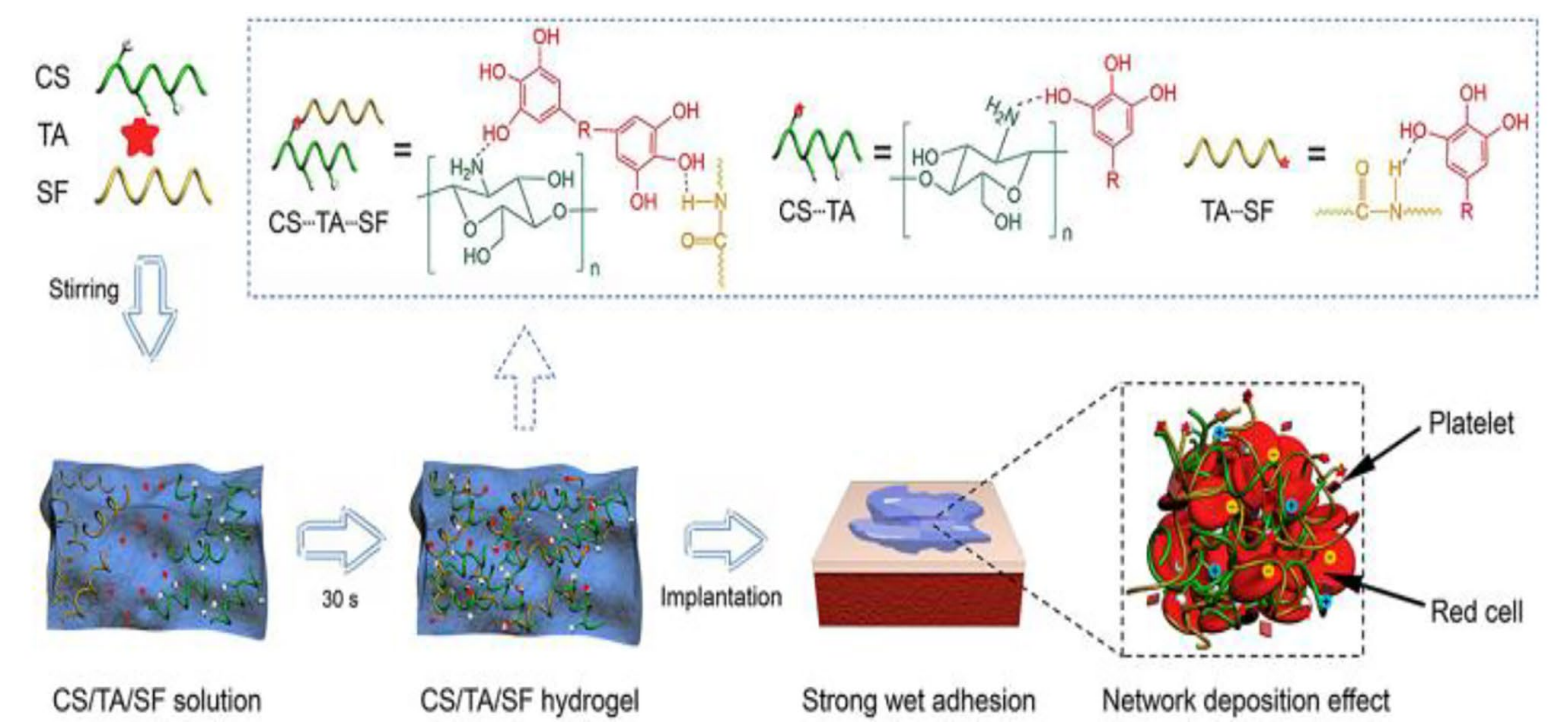
A diagram depicting how a mussel-inspired supramolecular hydrogel may provide a way for rescuers to achieve rapid hemostasis, especially when combined with chitosan (CS), silk fibroin (SF) and tannic acid (TA). *Reproduced with written permission from Qiao et al.* [22]

Building off the previous study, a team of South Korean researchers studied mussel adhesive protein (MAP). MAP is readily produced by the blue sea mussel (*Mytilus edulis*) and may have the potential to mitigate scarring. (Fig. 3) [24]. Wounds treated with MAP-based regimens exhibited superior reepithelialization, wound contraction, and neovascularization compared to controls in a rat skin excisional model [24]. Additionally, due to MAP’s ability to bind type I collagen, the substance possesses a novel ability to serve as a scar-preventive surgical glue [24]. Low-concentration uses of the black-lip pearl oyster (*Pinctada margaritifera*) have been shown to have similar effects in an in vitro toxicity assessment on epidermal and dermally derived cells [25].

## Limitations and potential complications

Although malaco-dermatology offers promising therapies, clinicians and researchers must remain vigilant of potential limitations. Allergic reactions to mollusk-based products may occur via consumption, inhalation, or cutaneous exposures leading to allergic or irritant contact dermatitis. Notably, the bodies of mollusks and invertebrates contain the tropomyosin protein, which is thought to be a significant allergen driver [26]. Additionally, certain cephalopod species produce harmful toxins, namely tetrodotoxin, a neurotoxin that may lead to human toxicity if tetrodotoxin-containing species and their products are not properly processed [27].

## Ethical considerations and summary

The field of dermato-malacology represents a fascinating convergence of marine biology and dermatology, offering a rich source of bioactive compounds with diverse therapeutic properties. Although the field captivates researchers and clinicians alike, ignoring the ethical considerations associated with these products’ discovery, mass production, and commercialization would be remiss. Since a large majority of mollusk-based product harvesting occurs overseas, particularly within Southeast Asia, little social or environmental impact data is available. Though extensive research and clinical testing are needed to implement these products, they have the potential to reshape dermatological care and advance our understanding of natural remedies derived from mollusks. We encourage researchers and clinicians to remain vigilant when utilizing any animal-based product.

## Data Availability

No datasets were generated or analysed during the current study.
